# Joint modeling of longitudinal change of fasting blood sugar and systolic blood pressure with survival time to death among hypertension patients

**DOI:** 10.1038/s41598-022-27120-y

**Published:** 2023-01-09

**Authors:** Markos Abiso Erango

**Affiliations:** Department of Statistics, College of Natural Sciences, Arba Minch University, Arba Minch, Ethiopia

**Keywords:** Biomarkers, Health care, Medical research, Risk factors

## Abstract

Hypertension is a universal public health challenge and a leading modifiable risk factor for cardiovascular disease and death. It is also called high blood pressure, described by two measured quantities systolic blood pressure (SBP) 140 mmHg or greater and diastolic blood pressure (DBP) 90 mmHg or greater. As the result, this study aims to use the joint model application to identify the factors that affect longitudinal changes in fasting blood sugar, SBP, and survival time to death of hypertension patients and their associations admitted to the Arba Minch General Hospital. We considered a total of 354 random samples of hypertension patients who had under follow-up at Arba Minch general hospital from January 2012 to February 2020. Among 2330 hypertension patients under follow-up, 354 were selected with a simple random sampling technique, and data was collected from the patient’s medical cards. After evaluating the longitudinal data with a linear mixed model and the baseline data with Cox proportional models, the joint models of both sub-models were assessed in R software version 4.2. According to the findings, the association between longitudinal changes (FBS, SBP, and time to death in hypertension patients was statistically significant. Ages, place of residence, lifestyle change, stages of hypertension, blood cholesterol level, related diseases, adherence to treatment, family history of hypertension patients, and DBP at baseline were associated factors that affect survival time and longitudinal measurement of FBS and SBP of the patients. The computed association parameters revealed subject-specific values. The subject-specific linear time slope of FBS and SBP was negatively related to the hazard rate of time to death of hypertension patients in Arba Minch general hospital. To reduce the risk of hypertension in patients, health professionals, governmental organizations, and non-governmental organizations must promote the implementation of community-based screening programs for early detection of hypertension.

## Introduction

Hypertensions one among the foremost common causes of premature death and morbidity and features a major impact on health care costs and it is a crucial public health challenge to both developed and developing countries. Hypertension is the biggest risk factor liable for 9.4 million deaths and illnesses worldwide and this makes it the only most vital explanation for morbidity and mortality worldwide^1,2^.

According to a systematic review conducted on awareness of hypertension in Africa, the levels of awareness, treatment, and control varied widely from country to country. Also, A study in Afghanistan and in sub-Saharan African countries such as Uganda, South African, Tanzania, and Nigeria) showed that hypertension the leading cause of morbidity and mortality^3,4^.

In Ethiopia different study showed that hypertension is growing health problem and it is one of risk factor for non-communicable diseases. Also meta-analysis on the prevalence of hypertension in Ethiopia found that it is increasing with an estimated prevalence of 19.6%. In the last few years, due to the lifestyle change from urbanization and demographic transition the burden of hypertension could be rise^5,6^.

Patients' health can be evaluated using both longitudinal and time-to-event data, and they frequently co-occur. The longitudinal trajectories might also be connected to the duration of the event. The results of separate analysis of longitudinal data and survival data may be inaccurate or biased. On the other hand, joint models of longitudinal and survival data take into account all available data at once and offer accurate and effective predictions^7^. This study aimed to use the joint model application to identify the factors that affect longitudinal changes in fasting blood sugar (FBP), SBP, and survival time to death of hypertension patients and their associations admitted to the Arba Minch General Hospital.

## Data and methods

### Study area

This study has been conducted on data taken from Arba Minch General Hospital (AMGH) which is located in South nation nationality people regional state Gamo zone, Arba Minch town, 750 km from Addis Ababa, Ethiopia. This hospital serves as a General hospital for the people in the surrounding area.

### Study design

To achieve the study’s objective, a retrospective study was done to collectimportant information from the medical files of patients with hypertension.

### Data source

To achieve the study’s objective, a retrospective study was done to collect important information from the medical files of patients with hypertension. The data for this study were collected from patients' medical cards, which contained epidemiological, laboratory, and clinical information regarding the patients who had been followed up at Arba Minch General Hospital from January 2012 to February 2020. The timing of the death of hypertension patients was the survival endpoint of interest.The research proposal of this study was checked and approved by Arba Minch University department of statistics and ethical clearance committee of the University. So, Informed consent has been waived by the ethical committee of the University. All study procedures were performed following relevant guidelines and regulations laid down by the Committee.

### Ethics declarations

The letter of ethical clearance was obtained from the institutional review board of Arba Minch University, and Arba Minch General Hospital gave permission to collect data of hypertension patients from recorded cards. For the purpose of confidentiality, there were no links with individual patients and all data had no personal identifier and were kept confidential and therefore Informed consent has been waived by Arba Minch University ethical committee.

### Study variables

The longitudinal changes in systolic blood pressure, fasting blood sugar and the survival time of hypertension patients after initiating medication were the study’s response variables. The longitudinal measurements were measured repeatedly over time for each hypertension patient under follow up. The survival time was measured time in months of a patient to the associated death event. Moreover, it is time to death event of the patient under the follow-up.

The Explanatory variables considered in this study were: observed time, square of observed time gender, base line age, place of residence, alcohol use, Khat intake, tobacco use, status of stress, stages of hypertension disease, life style change, blood cholesterol level, adherence to treatment, related diseases, family history of hypertension patients, DBP, diabetes of mellitus status were all factors considered.

### Statistical models

#### Statistical models

Different statistical models such as the joint model, the longitudinal linear mixed-effect (LME) model; and the event time model were investigated. The analysis was carried out using R software version 4.2.A linear mixed-effect model for longitudinal data and a Cox proportional hazard (PH) model for survival data, with an association parameter, show the effect of longitudinal measurement on hypertension patients survival time to death.

#### Linear mixed effect model for longitudinal measurements

The SBP and FBS measurements were collected longitudinally from the same individuals throughout several observation times. The LME model is a parametric linear model for longitudinal or repeated measurement data that quantifies the associations between a continuous dependent variable and a large number of predictor factors. It builds on the traditional linear regression model by accounting for both fixed and random factors. Subject-specific effects are included in the random effect^8^.

Let $y_{ijk}$ represent the $j th$ observation of the $k th$ outcome variable for the $i ^{th}$ subject, where:

i = 1, 2…354, j = 1, 2… $$n _{i}$$ and also, K = 1, 2 the vector ($$y_{1ik}$$ + $$y_{2ik} + \cdots +$$ $$y_{nik} )^{T}$$ represents the $$n_{ik}$$ observations of the *k*response variable from the $$ith$$ subject and vector ($$y_{1k}$$, $$y_{2k}$$,…$$y_{nik} )^{T}$$ represent the $$N_{k}$$ observation for the $$k th$$ response variable across all response variables and subjects, finally the vector ($$y_{1} y_{2}$$, $$y_{3}$$,…$$y_{n}$$)^T^ represents the observations across all response variables and subjects shown in (Eq. 1)^9^.

1 $$\left\{ {\begin{array}{*{20}l} {Y_{ijk} \left( {\text{t}} \right) = {\text{X}}_{{{\text{ik}}}} \left( {\text{t}} \right)\beta_{k} + {\text{Z}}_{{{\text{ik}}}} \left( {\text{t}} \right){\text{b}}_{{{\text{ik}}}} + {\upvarepsilon }_{{{\text{ik}}}} \left( t \right)} \hfill \\ {Y_{ij1} \left( {\text{t}} \right) = {\text{X}}_{{{\text{i}}1}} \left( {\text{t}} \right)\beta_{1} + {\text{Z}}_{{{\text{i}}1}} \left( {\text{t}} \right){\text{b}}_{{{\text{i}}1}} + {\upvarepsilon }_{{{\text{i}}1}} \left( t \right)} \hfill \\ {Y_{ij2} \left( {\text{t}} \right) = {\text{X}}_{{{\text{i}}2}} \left( {\text{t}} \right)\beta_{2} + {\text{Z}}_{{{\text{i}}2}} \left( {\text{t}} \right){\text{b}}_{{{\text{i}}2}} + {\upvarepsilon }_{{{\text{i}}2}} \left( t \right)} \hfill \\ {m_{ik} = {\text{X}}_{{{\text{ik}}}} \left( {t_{i} } \right)\beta_{k} + {\text{Z}}_{{\text{i}}} k\left( {\text{t}} \right){\text{b}}_{{{\text{ik}}}} } \hfill \\ {{\text{b}}_{{\text{ik }}} \sim {\text{ N}}\left( {0,{\mathbf{D}}} \right)} \hfill \\ {{\upvarepsilon }_{{{\text{ik}}}} \left( {\text{t}} \right)\sim {\text{N}}\left( {0,{\mathbf{R}}} \right)} \hfill \\ {{\text{ b}}_{1} \ldots ..{\text{b}}_{{\text{N}}} ,{\upvarepsilon }_{1} \ldots ..{\upvarepsilon }_{{\text{N}}} .{\text{independent}}} \hfill \\ \end{array} } \right\}$$

where$Y_{ijk} \left( {\text{t}} \right)$ is the corresponding true underlying longitudinal measures of the $kth$ biomarker for the $i\,th$ subject; jth observation, which is the ($n_{i}$) dimensional response matrix for subject $i$ 1 $\le i \le N, N$ is the number of subjects,${\text{X}}_{{{\text{ik}}}} \left( {\text{t}} \right)$ and ${\text{Z}}_{{{\text{ik}}}} \left( t \right)$ were the $1xp$ and $1xr (0 < r \le p)$ design matrix of fixed and random effects, respectively. The $px1$ and $rx1$ vectors of corresponding fixed and random effects parameters are $\beta_{k}$ and ${\varvec{b}}_{ik}$; $m_{ik}$ denotes the true, unobserved value of longitudinal response; and $\varepsilon_{ik}$(t) the measurement error.

#### Survival sub model

For the survival sub-model, we propose Cox PH and event time is the time elapsed up to the occurrence of an event of interest, which is death, given that it has not previously occurred yet.

Let $T^{*}_{i}$ and $C_{i}$ be the true event time and censoring time, respectively for subject $i$. The observed event time is defined as = min ($T_{i}^{*} ,C_{i}$) and the event indicator δi = I ($T_{i}^{*}$,$C_{i}$). Assuming that the hazard function depends on some functions of the true longitudinal measures $m_{ik} \left( {\text{t}} \right)$ and the baseline covariates, the hazard function^10^.

To achieve this we introduce the term $m_{ik} \left( {\text{t}} \right)$ that denotes the true and unobserved value of the longitudinal outcome at time ${\text{t}}$. Note that $m_{ik} \left( {\text{t}} \right)$ is different from $y_{ik} \left( {\text{t}} \right)$ with the latter being the contaminated with measurement error value of the longitudinal outcome at time ${\text{t}}$. To quantify the strength of the association between $m_{ik} \left( {\text{t}} \right)$ and the risk for an event, a straightforward approach is to postulate a PH models of the form (Eq. 2):

2 $$\begin{aligned} h_{i} \left( {t|M_{ik} \left( t \right),x_{i} } \right) = & \mathop {\lim }\limits_{dt \to 0} \frac{{\Pr \left( {t \le T_{i}^{*} < t + dt{|}T_{i}^{*} \ge t,M_{ik} \left( t \right),x_{i} } \right)}}{dt} \\ = & h_{o} \left( {t } \right)\exp \left( {\gamma^{T} X_{i} + \alpha_{k} m_{ik} \left( t \right)} \right),t > 0 \\ \end{aligned}$$

## Results and discussions

In this study, among 354 hypertension patients 54.52% were females and 45.48% were males. The majority of the patients were detected at the age of > 50. That means 51.13% patients older than 50 years and 48.87% the patients younger 50 years. Regarding the residence area of the patients, about 63% of patients were living in urban areas, and 37% of patients resided in rural areas with death proportions of 11.66%, and 6.87% respectively. From a total of 354 hypertension patients, 35 (10%) died, while 319 (90%) were censored (Table 1).

**Table 1:** Baseline categorical characteristics for hypertension patients at Arba Minch General Hospital from *January 2012 to* February*2020.*

Variables	Categories	Total (%)	Death proportion
Gender	Female	193 (54.50)	9.00
	Male	161 (45.50)	10.00
Age	$\le 50$	173 (48.87)	3.88
	$> 50$	181 (51.13)	16.18
Place of residence	Rural	131 (37.00)	6.87
	Urban	223 (63.00)	11.66
Alcohol use	No	218 (61.58)	5.96
	Yes	136 (38.42)	16.18
Khat intake	No	265 (74.86)	5.28
	Yes	89 (25.14)	23.60
Tobacco use	No	255 (72.00)	9.80
	Yes	99 (28.00)	10.10
Level of stress	No	252 (71.19)	9.52
	Yes	102 (28.81)	10.78
Stages of hypertension	Normal	29 (8.20)	3.45
	Elevated	51 (14.40)	5.88
	Stage_1_	87 (24.60)	6.89
	Stage_2_	116 (32.80)	9.48
	Hypertensive crisis	71 (20.00)	20.00
Life style modification	Yes	249 (70.34)	8.84
	No	105 (29.66)	12.38
Blood cholesterol level	Normal	247 (69.77)	4.45
	Raised	107 (30.23)	22.43
Adherence to treatment	Poor	223 (63.00)	11.66
	Good	131 (37.00)	6.87
Related diseases	None	168 (47.46)	1.79
	Stroke	42 (11.86)	35.71
	Heart case	95 (26.84)	20.00
	Others	49 (13.84)	4.08
Family history of patients	Negative	123 (34.75)	7.317
	Positive	231 (65.25)	11.25
Diabetes mellitus status	No	225 (63.56)	5.78
	Yes	129 (36.44)	17.05
Status	Censored	319 (90.00)	
	Event	35 (10.00)	

The baseline average mean of FBS, SBP, and DBP of patients were 132.47, 162.12 and 99.93, with a standard deviation of 54.91, 25.52 and 13.91, respectively; which means that an average SBP and DBP at baseline were different from the range of normal human blood pressure (Table 2).

**Table 2:** Baseline characteristics of continuous variables of the patients.

Variables	Mean	Median	SD
FBS	132.47	115.5	54.91
DBP	99.93	100	13.91
SBP	162.12	160	25.52

*NB FBS* Fasting blood sugar, *DBP* Diastolic blood pressure, *SBP* Systolic blood pressure.

Heterogeneous first order autoregressive variance–covariance has lowered Akaike information criteria (AIC), and Bayesian information criteria (BIC) values than those in other covariate structure, indicating that heterogeneous first order autoregressive variance–covariance is the best fit for our data when compare to the other covariance structures for the longitudinal change in SBPand FBSof hypertension data (Table 3).

**Table 3:** Comparison of the covariance structure of a linear mixed effect model.

Covariance structure	SBP		FBS	
	AIC	BIC	AIC	BIC
Compound symmetry	11631.6	11789.7	7780.5	7893.3
Heterogeneous compound symmetry	11662.3	11720.7	7782.4	7861.1
Heterogeneous first order autoregressive	10689.1	11145.8	7090.5	7560.3
Unstructured	11327.8	11404.8	7248.1	7761.9

*AIC* Akaike information criteria, *BIC* Bayesian information criteria, *SBP* Systolic blood pressure, *FBS* Fasting blood sugar.

The model with random intercept and slope was chosen as the most parsimonious model for the LME model for the longitudinal change of SBPandFBSdue to lower values of AIC and BIC for both FBS and SBP (Table 4).

**Table 4:** Selection of random effects to be included in the linear mixed effect model for FBS and SBP hypertension patients data.

Models for random effect	SBP		FBS	
	AIC	BIC	AIC	BIC
Random intercept model	11072.87	11626.8	7156.7	7512.5
Random intercept and slope model	10675.1	11454.3	7072.6	7420.1
Random intercept and quadratic slope	11498.3	11750.0	7834.2	8082.0

*AIC* Akaike information criteria, *BIC* Bayesian information criteria, *SBP* Systolic blood pressure, *FBS* Fasting blood sugar.

The association parameters $r_{1 }$ and $r_{2}$ are statistically significant ($\hat{r}_{1}$ = − 1.05 with *p*-value < 0.009 and $r_{2 } = - 0.035$) with *p*-value < 0.001 respectively. The significance of the association parameter suggests that there is a strong dependence (association) between longitudinal changes (SBP and FBS) with survival time of hypertension patients.

The joint model analysis in Table 5 below showed that the longitudinal changes of (SBP and FBS) were significantly associated with observed time, square of observed time, place of residence, alcohol use, life style change, tobacco use, age, blood cholesterol level, adherence to treatment, family history of hypertension patient. Furthermore, in the survival sub-model, the survival time of hypertension patients was related to baseline age, place of residence, life-style change, Khat intake, stages of hypertension diseases, blood cholesterol level, related diseases, adherenceto treatment, family history of hypertension and diastolic blood pressure measurement at base line.

**Table 5:** Joint model analysis of FBS, SBP and the time to death of hypertension patient’s data at AMGH.

Longitudinal sub-model					
Parameters	Category	SBP		FBS	
		$\widehat{ \beta }$	P-value	$\hat{\beta }$	*p*-value
Intercept		0.37	0.002	11.112	0.001
Observed time		− 0.41	0.002	− 0.002	0.035
Observed time square		0.015	0.0018	1.060	0.007
Gender (ref = female)	Male	0.026	0.019	2.511	0.015
Place of resident (ref = rural)	Urban	0.36	0.009	2.665	0.010
Alcohol use (ref = No)	Yes	0.045	0.003	0.064	0.038
Tobacco use (ref = No)	Yes	− 0.063	0.0001	0.278	0.016
Khat use (ref = No)	Yes	0.039	0.0009	0.086	0.043
Age (ref = $\le 50)$	> 50	− 0.32	0.071	7.299	0.002
Diabetes mellitus status (ref = No)	Yes	− 0.022	0.058	− 1.257	0.062
Status of stress (ref = No)	Yes	0.028	0.092	0.165	0.034
Life style change (ref = Yes)	No	0.020	0.0001	4.22	0.030
Adherence (ref = good)	Poor	0.215	0.0002	0.084	0.009
Cholesterol level (ref = Normal)	Raised	0.021	0.0001	3.340	0.010
Stage of hypertension (ref = Normal)	Elevated	10.570	0.016	0.186	0.002
	Stage 1	12.150	0.019	0.187	0.015
	Stage 2	17.360	0.015	0.152	0.046
	Hypertensive crisis	17.730	0.018	0.385	0.002
Family history (ref = Positive)	Negative	− 0.57	0.0001	− 6.764	0.009

Survival sub-model					
Parameters	Category	$\hat{\user2{\beta }}$	HR	*p*-value	
Intercept		2.170	8.758	0.002	
Gender (ref = female)	Male	− 0.013	0.987	0.000	
Age (ref = $\le 50$)	> 50	0.270	1.027	0.000	
Alcohol use (ref = No)	Yes	0.140	1.150	0.001	
Khat use (ref = No)	Yes	0.170	1.180	0.002	
Lifestyle change (ref = Yes)	No	0.297	1.346	0.007	
Place of resident (ref = rural)	Urban	0.015	1.015	0.000	
Related diseases (ref = None)	Stroke	5.700	298.860	0.002	
	Heart case	2.500	12.428	0.005	
	Other	− 3.922	0.020	0.004	
Tobacco use (ref = No)	Yes	0.220	1.246	0.001	
blood cholesterol level (ref = Normal)	Raised	0.018	1.018	0.001	
Family history (ref = Positive)	Negative	− 0.127	0.881	0.002	
Diabetes status (ref = No)	Yes	0.190	1.210	0.002	
Adherence (ref = Good)	poor	0.221	1.247	0.001	
Stage of hypertension (ref = Normal)	Elevated	0.163	1.178	0.001	
	Stage 1	− 0.057	0.945	0.002	
	Stage 2	0.254	1.289	0.003	
	Hypertensive crisis	0.278	1.320	0.003	
DBP at baseline		0.010	1.010	0.002	
**Association parameters**					
$r_{1}$ (SBP)		− 1.05		0.009	
$r_{2}$ (FBS)		− 0.035		0.001	

For instant, in a survival sub-model, all the estimated average regression coefficients, hazard ratio and up values off baseline age, lifestyle change, Khat use, blood cholesterol level and adherence to treatment are 0.27, HR = 1.027 (*p* < 0.002), 0.297, HR = 1.346 (*p* < 0.007), 0.17, HR = 1.18 (*p* < 0.002), 0.0183HR = 1.018 (*p* < 0.0015), and 0.221, HR = 1.247 (*p* < 0.001) were significant effect respectively at 5%level of significance (Table 5).These estimates show that, while an increase in age of the patients increases the hazard rate of death, patients who have no lifestyle change have higher hazard to death. Similar, patients who use Khat and tobacco have higher rates of death compared to patients who did not use Khat and tobacco. This shows that Khat and Tobacco negatively influence the survival time of hypertension patients.

The stages of hypertension disease increases (from normal stage to hypertensive crisis stage) the survival time of hypertension patients’ decrease. And an increase in raised blood cholesterol level, poor adherence to treatment, related diseases, positive family history of hypertension patients, base line FBS level measurement, base line diastolic blood pressure measurement respectively increases hazard to death although the survival time of patient declines.

## Discussion

The purpose of this study was to use the joint model application to identify the factors that affect longitudinal changes in SBP, FBS, and survival time to death of hypertension patients and their associations admitted to the Arba Minch General Hospital. In this study, the founding from the analysis of the survival sub-model shows that variables like age, alcohol use, Khat use, lifestyle change tobacco use, blood cholesterol level, family history, diabetic status, and stage of hypertension were significantly associated with survival time of the patients. The study revealed that the variables place of residence and observation time had significant associations with changes in SBP. This finding was similar to the finding in Jimma University specialized hospital, south-west Ethiopia reported that place of residence, and observation time have significant effects on change in SBP measurement^11^. Also, the current study reveals that the stage of hypertension, family history of adherence to treatment, related disease, and smoking tobacco are identified as risk factors that affect the longitudinal change (SBP and FBS) and survival time of the patients. This finding is similar to the finding of a study in Uganda^12^. But, this result was contradictory to research conducted in eastern Ethiopia^13^.In this study, smoking tobacco, Khat use, and alcohol use were significantly associated with survival time of hypertension. The study reveals that patients who did not use alcohol did not use Khat and did not smoke tobacco had a better survival probability than those who used alcohol, Khat and smoking tobacco. Similarly, Ayalew et al. indicated that alcohol use is a potential risk factor for hypertension, and they also found hypertension was significantly higher in individuals who use alcohol than in those who did not use it^14^. This study finds that there is a strong dependence between SBP, FBS, and survival time of hypertension patients at AMGH, and suggests that joint model analysis rather than separate model analysis for such kind of dependence outcomes, clinical and medical studies. These findings are agreed with previous researchers’ findings^15–17^. In this study, the finding regarding the relationship between place of residence of patients and survival time until hypertension associated death was similar with finding in Addis Ababa Ethiopia reported that place of residence has a significant effect on hypertension patients' survival time^14^. Moreover, we found that a family history of hypertension disease was a statistically significant risk factor for death in hypertension patients; this finding is consistent with the findings of other researchers^18^. The current study found that age was related to the survival time of hypertension patients. This result is consistent with findings from a study in Ethiopia and Uganda^12,14^. Our study found that Khat intake was associated with the survival time of hypertension patients. These results are consistent with findings from a study^19^ that identified that Khat chewing is one of the risk factors for hypertension mortality.

## Conclusions

The linear mixed-effect model with subjective values of SBP and FBS was a good fit for the longitudinal changes in SBP and FBS evaluations in this investigation. At a 5% level of significance, the variables including observed time, square of observed time, age, place of residence, alcohol use, lifestyle change, tobacco use, blood cholesterol level, adherence to treatment, family history, and diastolic blood pressure were significantly associated with longitudinal measurement of (SBP and FBS) Joint models were used with the assumptions of a linear mixed effect model for the longitudinal change of (SBP and FBs) with survival time of hypertension patients data collected from AMGH. Model comparison was employed to select fitted model, Weibull parametric model was fitted model for survival analysis. In survival sub-model covariates: baseline age, place of residence, lifestyle change, Khat intake, stages of hypertension disease, blood cholesterol level, related diseases, adherence to treatment, family history, and baseline diastolic blood pressure measurement were significantly affect the survival time of hypertension patients at 5% level of significance. In joint model analysis, association parameters were significant at a 5% level of significance. Thisindicates that there is a strong dependence between longitudinal changes (SBP and FBS) and with survival time of patients. The negative sign of the estimated association parameter indicates longitudinal changes (SBP and FBS) measurementwere negatively associated with the survival time of the patients in the study area, implying lower values of the SBP and FBS measurements associated with better survival time of the patients. Based on the findings, we recommended that patients in societies who have positive family history should take action on early detection and prevention mechanisms as well as should be aware of the risk factors. Also for further improvements, the Policymakers, concerned bodies, and Arba Minch General Hospital should give more attention and intervention to identified risk factors in society, and Programs should design to improve the surveillance systems and implementations of community-based screening programs for early detection of hypertension are recommended.

## Data Availability

The dataset used or analyzed during the current study available from the corresponding author on reasonable request.
